# Chronic Copper Overload Triggers Inflammation in Mesenteric PVAT Alongside Changes in Renin–Angiotensin System-Related Pathways

**DOI:** 10.3390/nu17132082

**Published:** 2025-06-23

**Authors:** Nina Bruna de Souza Mawandji, Nayara Ariel da Silva Lisboa, Karoline Neumann Gomes, Júlia Martins Vieira, Jussara de Jesus Simão, Maria Isabel Alonso-Vale, Karolini Zuqui Nunes, Dalton Valentim Vassallo, Andressa Bolsoni-Lopes

**Affiliations:** 1 Postgraduate Program in Physiology Sciences, Health Sciences Center, Federal University of Espirito Santo, Vitoria 29043-900, ES, Brazil; ninamawandji@gmail.com (N.B.d.S.M.);; 2 Postgraduate Program in Nutrition and Health, Health Sciences Center, Federal University of Espirito Santo, Vitoria 29043-900, ES, Brazil; 3 Postgraduate Program in Chemical Biology, Institute of Environmental Sciences, Chemical and Pharmaceutical, Federal University of São Paulo, Sao Paulo 04039-032, SP, Brazilalonso.vale@unifesp.br (M.I.A.-V.); 4 Postgraduate Program in Nutrition, Paulista School of Medicine, Federal University of São Paulo, Sao Paulo 04039-032, SP, Brazil

**Keywords:** copper, adipose tissue, inflammation, adipokines, renin–angiotensin system, receptor angiotensin type 1

## Abstract

**Background/Objectives:** Copper is an essential micronutrient required for physiological functions, but elevated serum levels impair vascular reactivity and blood pressure regulation. Given PVAT’s critical role in vascular function, this study aimed to investigate the effects of chronic copper overload on the secretory function of mesenteric PVAT, focusing on its vasoregulatory role. **Methods:** In the first phase, 8-week-old male Wistar rats were assigned to two groups, namely control (saline, i.p.) or copper (25.72 µg/kg/day Cu, i.p., for 30 days), corresponding to twice the recommended daily dose of copper. In the second phase, rats were divided into four groups: control (saline, i.p., water by gavage), copper (Cu, i.p., water by gavage), losartan (saline, i.p., 10 mg/kg/day losartan by gavage), or copper + losartan (Cu, i.p., 10 mg/kg/day losartan by gavage). After euthanasia, mesenteric PVAT was collected for morphometric analysis, gene and protein expression of adipokines, inflammatory molecules, and the renin–angiotensin system. Serum was used for hormone and biochemical measurements. **Results:** In mesenteric PVAT, chronic copper overload increased adipocyte diameter and reduced lipolysis. It also elevated the secretion of TNF-α and PAI-1 while decreasing IL-10 levels. Additionally, it upregulated the mRNA expression of *MCP-1*, *F4/80*, *CD86*, *TLR4*, *arginase-1*, *iNOS*, *ACE1*, and *AT1R*, alongside an increase in serum angiotensin II levels. When copper treatment was combined with losartan, an AT1R antagonist, adipocyte hypertrophy; TNF-α secretion; and the gene expression of *TLR4*, *F4/80*, and *arginase-1* were attenuated. **Conclusions:** Chronic exposure to double the recommended dose of Cu disrupts the secretory function of mesenteric PVAT, promoting inflammation and altering the local RAS. These effects appear to occur, at least in part, alongside the activation of the AT1R–TLR4–angiotensin II signaling pathway, triggering the upregulation of vasoregulatory inflammatory markers.

## 1. Introduction

Copper is an essential micronutrient required for the body’s development and critical physiological functions. It acts as a cofactor or integral component of several enzymes, being directly involved in catalytic reactions related to energy metabolism, immune defense, and neuropeptide synthesis [1]. As a non-endogenous metallic element, copper intake occurs through food, water, medications, and dietary supplements [2,3]. With the growing availability of copper-containing supplements, often used without medical supervision, concerns have emerged regarding potential overexposure. When taken indiscriminately, copper supplementation can easily exceed the oral reference dose, increasing the risk of toxicity [4]. Although recommended intake levels vary across populations, the World Health Organization suggests a daily intake of 12.85 µg/kg [5].

Despite its beneficial effects, copper overload can generate reactive oxygen species (ROS), promote inflammation, and cause cellular damage [6,7,8]. Experimental studies have shown that acute exposure to high concentrations of copper (10 µg/mL) can lead to cardiovascular dysfunction, such as reduced papillary muscle contraction mediated by oxidative stress [9]. In addition, chronic exposure to copper (25.72 µg/kg/day) has been shown to impair vascular reactivity of the thoracic aorta in response to phenylephrine and increase inflammatory factors in thoracic PVAT [10,11]. Furthermore, prolonged exposure to elevated copper levels for 30 days in 12-week-old Wistar rats has been associated with increased systolic blood pressure [12].

Perivascular adipose tissue (PVAT) is the tissue attached to the tunica adventitia of blood vessels, except for cerebral and pulmonary vessels [13,14]. It is recognized as a metabolic and endocrine organ that significantly influences vascular structure and function through vasodilatory factors, such as nitric oxide (NO) and leptin, and vasoconstrictor factors, such as angiotensin II and resistin [15,16,17,18,19].

The phenotypic and functional characteristics of PVAT vary according to the location and heterogeneity of the vascular bed, which, together with adipocyte plasticity, determine its classification as white, beige, or brown adipose tissue [20,21]. Among these types, mesenteric PVAT is predominantly white, exhibiting high metabolic activity, particularly in lipolysis and inflammatory cytokine production [18,22,23]. Additionally, it plays a key role in regulating peripheral vascular resistance and systemic blood pressure [20,21]. Under physiological conditions, mesenteric PVAT maintains endocrine balance [24]. However, PVAT dysfunction can lead to inflammation, contributing to vascular damage through factors like the renin–angiotensin system (RAS) and inflammatory adipokines [15,19,24,25,26].

Although the adverse vascular effects of copper overload, including alterations in vascular reactivity and blood pressure regulation, are well established, its effect on mesenteric PVAT, a pivotal modulator of vascular homeostasis, remains unexplored. Therefore, this study aims to investigate the effects of chronic copper overload on the secretory function of mesenteric PVAT, with a focus on its vasoregulatory role.

## 2. Materials and Methods

### 2.1. Animals and Treatment

All experimental protocols were approved by the Animal Care Committee of the Health Sciences Center, Federal University of Espírito Santo (UFES), Brazil (#01/2023/CEUA). Male 8-wk-old *Wistar* rats (from the Animal Facility of the UFES) were randomly assigned to individual cages (n = 4 per group), acclimated for 7 days before the beginning of the protocol, at 23 °C on a 12:12 h light–dark cycle with food (balanced chow pellet diet; Nu-83 vilab CR1, Nuvital, Colombo, PR, Brazil) and water ad libitum.

In the initial phase of the experiments, rats were randomly assigned to one of two groups: control, in which rats received an intraperitoneal saline solution, and copper (Cu), in which rats received copper at 25.72 µg/kg/day intraperitoneally (copper chloride CuCl2; SIGMA-Aldrich, St. Louis, MO, USA; catalog number 222011, Lot BCBN9751V), over a 30-day treatment period. Subsequently, in the second phase, rats were randomly assigned to one of four groups: control, in which rats received an intraperitoneal saline solution and water by gavage; copper (Cu), in which rats received copper at 25.72 µg/kg/day intraperitoneally and water by gavage; losartan (Los), in which rats received an intraperitoneal saline solution and losartan at 10 mg/kg/day by gavage (EMS, Hortolandia, Brazil; catalog number 1023508100180); or copper + losartan (Cu + Los), in which rats received copper at 25.72 µg/kg/day intraperitoneally and losartan at 10 mg/kg/day by gavage, over a 30-day treatment period.

For the experimental model, we induced copper overload by administering twice the recommended daily intake, which is 12.85 µg/kg/day, as established by the Institute of Medicine [27] and the World Health Organization [5,11].

### 2.2. Experimental Procedure

Body weight and food intake were measured twice a week throughout the experiment. Food efficiency was calculated by the ratio of body weight gain (g) to food ingestion (g).

After 30 days, rats (8 h fasted) were anesthetized with Halothane and euthanized by exsanguination via cardiac puncture. Blood samples were centrifuged at 3500 rpm for 15 min at 4 °C, and serum was stored at −80 °C. Adipose fat pads (inguinal, visceral, and perivascular adipose tissue of the mesenteric artery) were harvested, weighed, and processed as described below.

### 2.3. Adipocyte Isolation

Adipocyte isolation was performed for diameter analysis as previously described [28], with some slight modifications [29]. Briefly, fat pads were minced in a flask containing DMEM supplemented with HEPES (20 mM), sodium pyruvate (2 mM), bovine serum albumin (BSA, 1%), and collagenase type 2 (1 mg/mL), pH 7.4, and incubated for 40 min at 37 °C in an orbital shaker. Isolated adipocytes were filtered through a plastic mesh (150 μm) and washed three times in the same buffer without collagenase. After washing, the medium was thoroughly aspirated, and adipocytes were harvested. The adipocytes were photographed under an optic microscope (×100 magnification) using a microscope camera (Moticam 1000; Motic, Richmond, BC, Canada), and the mean adipocyte diameter was determined by measuring 50 cells using Motic-Images Plus 2.0 software.

### 2.4. Plasma Biochemical Analysis

Blood glucose, triglyceride, and total cholesterol levels were determined using commercial kits (Labtest Diagnostica, Lagoa Santa, Brazil). Angiotensin II was quantified by ELISA (E-EL-R1430 Elabscience^®^ Biotechnology from Houston, TX, USA). The measurements were performed using a microplate photometer (Thermo Scientific Multiskan FC from Waltham, MA, USA).

### 2.5. Adipokine and Hormone Analyses

A fragment of mesenteric perivascular adipose tissue was incubated for 20 h in a culture plate with Dulbecco’s modified Eagle medium (DMEM-Invitrogen Life Technologies, Waltham, MA, USA; catalog number 41965062) containing glucose 4500 mg/L, 10% fetal bovine serum (Invitrogen Life Technologies, Waltham, MA, USA, catalog number A5256701) and penicillin–streptozotocin 1% (Nova Biotecnologia, São Paulo, Brazil; catalog number BR30110-01), pH 7.2, at 37 °C in a humidified 5% CO_2_ atmosphere. After incubation, the culture medium was collected and used for the quantification of adipokines using ELISA kits. Adiponectin, IL-10, and TNF-α were measured using ELISA kits from R&D Systems (Minneapolis, MN, USA; DYE100-05, DY522-05, and DYE510-05, respectively). PAI-1 and resistin were measured using ELISA kits from Elabscience^®^ (Houston, TX, USA; E-EL-R3025 and E-EL-R0614, respectively). Angiotensin II was quantified by ELISA (E-EL-R1430 Elabscience^®^ Biotechnology, Houston, TX, USA). The assay was performed according to the manufacturer’s protocols.

### 2.6. RNA Extraction and Quantitative Real-Time Polymerase

The total RNA was extracted from mesenteric PVAT, using Trizol reagent (Invitrogen Life Technologies, Waltham, MA, USA; Catalog number 15596026). The total RNA was analyzed for quality on ratios 260/280 and 260/230 nm on NANODROP (Thermo Scientific, Waltham, MA USA), and reverse-transcribed to cDNA using the High-Capacity cDNA Reverse Transcription Kit with RNase Inhibitor (Thermo Scientific, EUA, Waltham, MA, USA). Gene expression was assessed by chain reaction quantitative real-time polymerase (PCR) using a QuantStudio™3 Real-Time PCR System (Thermo Scientific) and fluorescent dye such as SYBR Green. Analysis of real-time PCR data was performed using the 2^−ΔΔCT^ method [30]. Data are expressed as the ratio of target gene expression to the housekeeping gene (B-actin). The primers used are shown in Table 1.

### 2.7. Lipolysis

Lipolysis was estimated by measuring the rate of glycerol release into the incubation medium. For this, isolated mesenteric adipocytes were incubated in Krebs–Ringer phosphate buffer (pH 7.4) containing BSA (20 mM) and glucose (5 mM) for 30 min at 37 °C in the presence or absence of isoproterenol (2 × 10^−6^ M). The reaction was stopped on ice, and the medium was carefully collected for measurement of glycerol release (Free Glycerol Determination Kit, SIGMA-Aldrich, St. Louis, MO, USA). Results are expressed as micromoles of glycerol per 1 × 10^6^ cells.

### 2.8. Statistical Analysis

Data are expressed as mean ± standard error of the average (SEM). Comparisons between the two groups were analyzed using Student’s *t*-test. Comparisons between three or more groups were performed using one-way ANOVA, followed by Tukey’s post hoc test. Differences were considered significant for *p* < 0.05. Statistical analysis was performed using GraphPad Prism software version 9.1.2 (GraphPad Software Inc., San Diego, CA, USA).

## 3. Results

### 3.1. Chronic Copper Overload Increases Mesenteric PVAT Adipocyte Diameter and Decreases Basal Lipolysis Without Altering Total Adiposity

The treatment of Wistar rats with copper overload (25.72 µg/kg/day) for 30 days did not alter body weight gain, feed efficiency, or the weight of visceral and inguinal subcutaneous white adipose tissue. However, it increased the mesenteric PVAT adipocyte diameter and reduced food intake. Additionally, there were no changes in triglyceridemia, cholesterol levels, or blood glucose, as shown in Table 2.

**Figure 1:**
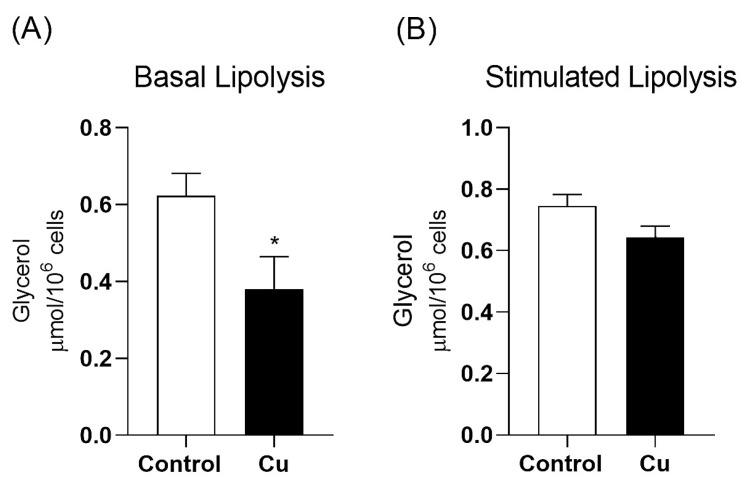
Basal (Panel (**A**)) and isoproterenol-stimulated (Panel (**B**)) glycerol release rates in adipocytes isolated from mesenteric PVAT of Wistar rats treated for 30 days with daily intraperitoneal injections of saline (control) or copper (25.72 µg/kg/day). n = 7 per group. Data are expressed as mean ± SEM. * p < 0.05 vs. control (Student’s *t*-test). Cu: copper.

Among investigations into the metabolic regulation of adipocytes, chronic copper treatment was found to significantly decrease basal lipolysis, as indicated by the lower rates of glycerol release into the medium from adipocytes of the Cu group compared to the control group (0.38 ± 0.08 and 0.62 ± 0.05 µmol/10^6^ cells, respectively. No changes were observed in glycerol release during isoproterenol-stimulated lipolysis (Cu = 0.64 ± 0.03; control = 0.74 ± 0.02 µmol/10^6^ cells) (Figure 1).

### 3.2. Chronic Copper Overload Impairs the Inflammatory and Vascular Regulatory Adipokines of Mesenteric PVAT

**Figure 2:**
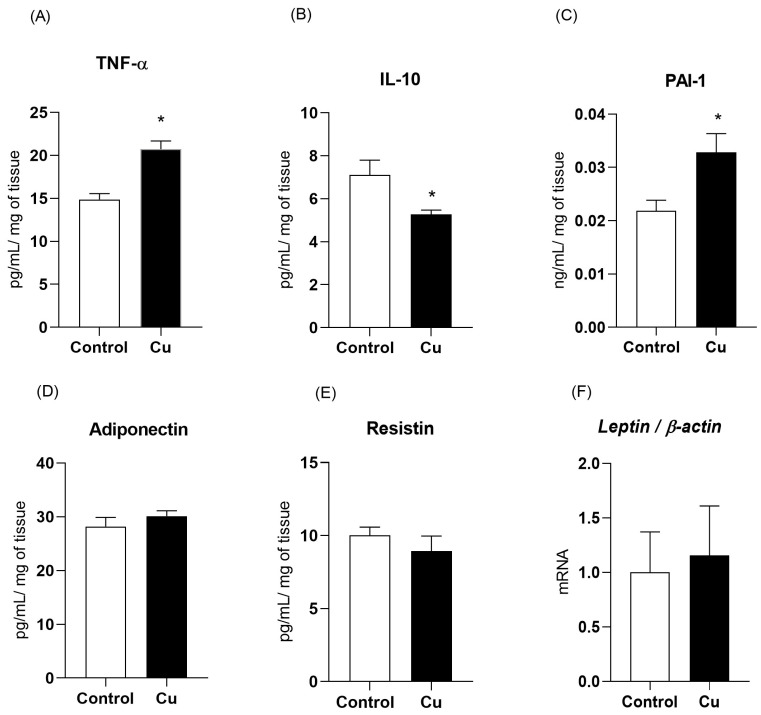
Adipokine secretion by the perivascular adipose tissue of the mesenteric artery. Tumor necrosis factor alpha (TNF-α—Panel (**A**)), interleukin-10 (IL-10—Panel (**B**)), plasminogen activator inhibitor type 1 (PAI-1, Panel (**C**), adiponectin (Panel (**D**)), resistin (Panel (**E**)), and *leptin* messenger RNA levels (Panel (**F**)) in Wistar rats treated for 30 days with daily intraperitoneal injections of saline (control) or copper (25.72 µg/kg/day). n = 8 per group. Data are expressed as mean ± SEM. * *p* < 0.05 vs. control. Student’s *t*-test. Cu: copper.

Regarding the secretory function of mesenteric PVAT, a comparison between the two groups indicated a 39% increase in tumor necrosis factor alpha (TNF-α) secretion and a 50.4% increase in PAI-1 in copper-treated rats, followed by a 35% decrease in IL-10. No differences were found in adiponectin (control = 28.2 ± 1.6; Cu = 30.12 ± 0.98 pg/mL/mg) or resistin secretion (control = 10 ± 0.5; Cu = 8.9 ± 1 pg/mL/mg) and *leptin* gene expression (control = 1 ± 0.3; Cu = 1.15 ± 0.4) (Figure 2).

**Figure 3:**
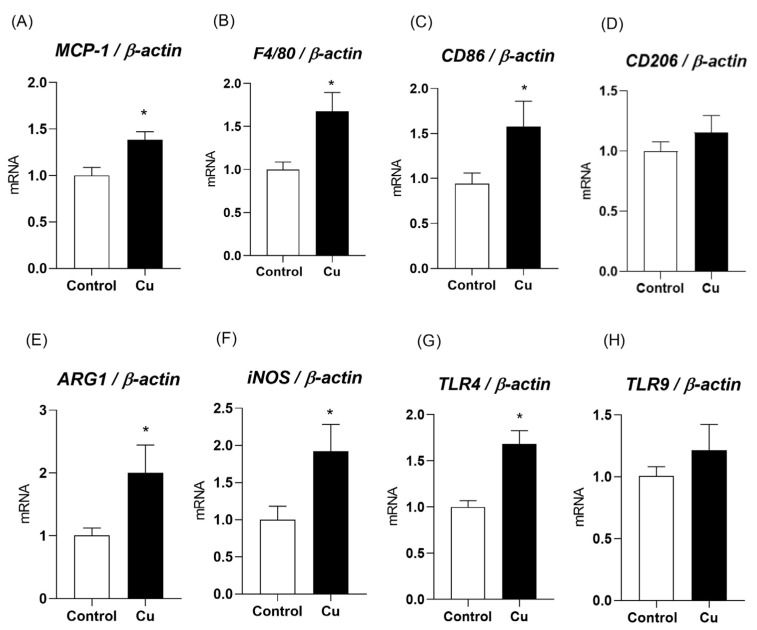
Messenger RNA levels of *monocyte chemoattractant protein-1* (*MCP-1*—Panel (**A**)), *F4/80* (Panel (**B**)), *CD86* (Panel (**C**)), *CD206* (Panel (**D**)), arginase-1 (*ARG-1*—Panel (**E**)), inducible nitric oxide synthase (*iNOS*—Panel (**F**)), *Toll-like receptor 4* (*TLR4*—Panel (**G**)), and *Toll-like receptor 9* (*TLR9*—Panel (**H**)) in the perivascular adipose tissue of the mesenteric artery of Wistar rats treated for 30 days with daily intraperitoneal injections of saline (control) or copper (25.72 µg/kg/day). n = 8 per group. Data are expressed as mean ± SEM. * *p* < 0.05 vs. control. Student’s *t*-test. Cu: copper.

Aiming to expand investigations regarding the secretory function of mesenteric PVAT, other proteins involved in canonical inflammatory pathways were evaluated. In copper-treated animals, an increase in the gene expression of *MCP-1* (control = 1.0 ± 0.08; copper = 1.38 ± 0.08), *F4/80* (control = 1.0 ± 0.08; copper = 1.67 ± 0.21), *CD86* (control = 1.0 ± 0.12; copper = 1.67 ± 0.3), *arginase-1* (control = 1.0 ± 0.11; copper = 2 ± 0.44), *iNOS* (control = 1.0 ± 0.18; copper = 1.9 ± 0.36), and the *TLR4* receptor (control = 1.0 ± 0.06; copper = 1.68 ± 0.14) was identified. No significant differences were detected in the gene expression of the *CD206* marker (control = 1.0 ± 0.07; copper = 1.15 ± 0.14) or *TLR9* (control = 1.0 ± 0.07; copper = 1.21 ± 0.2), as shown in Figure 3.

### 3.3. Chronic Copper Overload Promotes an Increase in Blood Angiotensin II Levels and Upregulation of Key Proteins in the Renin–Angiotensin System of Mesenteric PVAT

**Figure 4:**
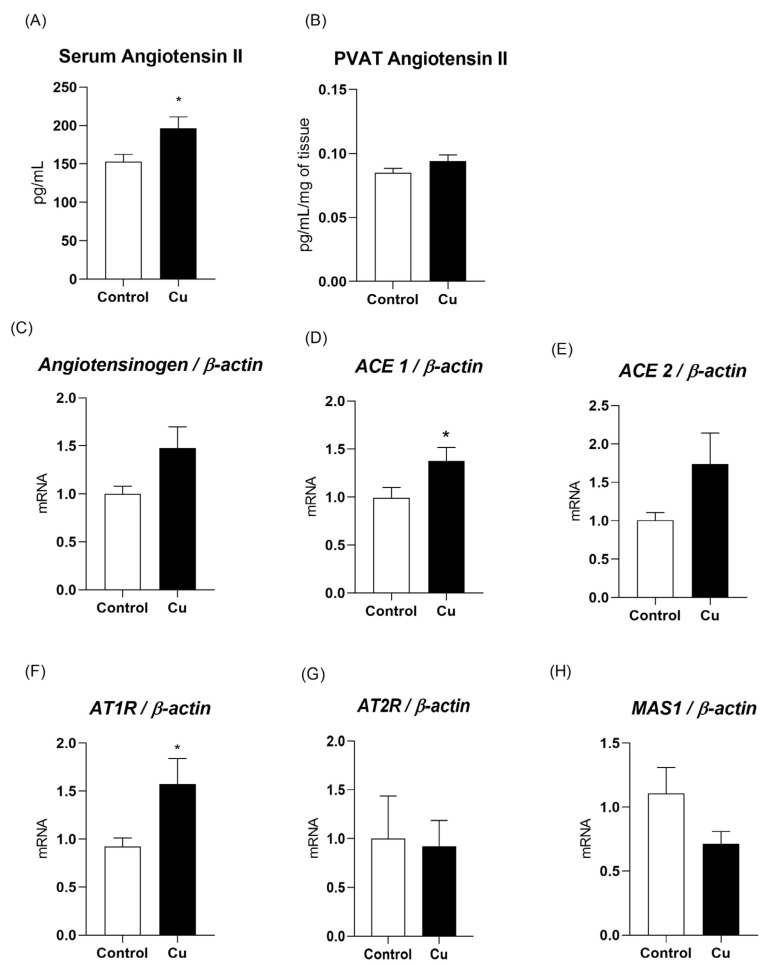
Serum angiotensin II (Panel (**A**)) and mesenteric PVAT angiotensin II levels (Panel (**B**)); messenger RNA levels of *angiotensinogen* (Panel (**C**)), *angiotensin-converting enzyme 1 (ACE1*—Panel (**D**)), *angiotensin-converting enzyme 2 (ACE2*—Panel (**E**)), *angiotensin II type 1 receptor (AT1R*—Panel (**F**)), *angiotensin II type 2 receptor (AT2R*—Panel (**G**)), and *MAS1 receptor* (Panel (**H**)) in the perivascular adipose tissue of the mesenteric artery of Wistar rats treated for 30 days with daily intraperitoneal injections of saline (control) or copper (25.72 µg/kg/day). n = 6–8 per group. Data are expressed as mean ± SEM. * *p* < 0.05 vs. control. Student’s *t*-test. Cu: copper.

An elevation in blood serum angiotensin II levels was identified in copper-treated animals (control = 153 ± 9.4; copper = 196.3 ± 14.9 pg/mL; Figure 4A). Although this increase was not observed in the isolated PVAT (control = 0.084 ± 0.003; copper = 0.094 ± 0.004 pg/mL/mg of tissue; Figure 4B), significant increases were found in the messenger RNA expression of *ACE1* (38%; Figure 4D) and *AT1R* (57%; Figure 4F) in the mesenteric PVAT of the copper group compared to the control group. Copper exposure did not alter the gene expression of *angiotensinogen*, *ACE2*, *AT2R*, *or MAS1* in mesenteric PVAT (Figure 4).

In an attempt to enhance the significance and relevance of these findings, we also investigated whether the changes in the secretory function of mesenteric PVAT toward a pro-inflammatory profile, induced by copper overload, involved mechanisms associated with the upregulation of AT1R from the PVAT renin–angiotensin system. To address this, we used copper treatment combined with losartan, an antagonist of the angiotensin II type 1 receptor.

### 3.4. The Effects of Copper Overload on Inflammatory Markers in Mesenteric PVAT Are Partially Prevented by Losartan

Similarly to the data presented in Table 2, the treatment of Wistar rats with copper overload (25.72 µg/kg/day) or copper combined with losartan (25.72 µg/kg/day + 10 mg/kg, respectively) for 30 days did not affect body weight gain, food efficiency, visceral white adipose tissue, inguinal subcutaneous adipose tissue, triglyceridemia, cholesterol levels, or blood glucose. However, the copper group, but not the copper + losartan group, exhibited an increase in the diameter of mesenteric adipocytes (Table 3).

**Figure 5:**
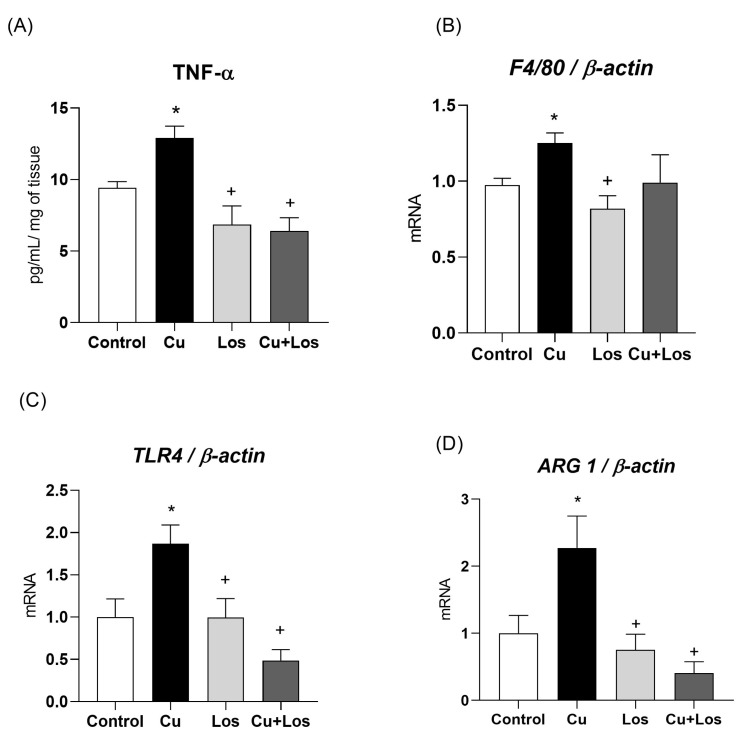
Secretion of tumor necrosis factor alpha (TNF-α—Panel (**A**)) and messenger RNA levels of F4/80 (Panel (**B**)), *Toll-like receptor 4 (TLR4*—Panel (**C**)), and *arginase-1 (ARG1*—Panel (**D**)) in the perivascular adipose tissue of the mesenteric artery of Wistar rats treated for 30 days with saline (control), copper (25.72 µg/kg/day), losartan (10 mg/kg), and copper combined with losartan (25.72 µg/kg/day; 10 mg/kg). n = 6 per group. Data are expressed as mean ± SEM. * *p* < 0.05 vs. control; + *p* < 0.05 vs. copper. One-way ANOVA followed by Tukey’s post hoc test. Ct: control; Cu: copper; Los: losartan; Cu + Los: copper combined with losartan.

Animals treated with copper showed higher TNF-α secretion (17.7 ± 0.8 pg/mL/mg) in mesenteric PVAT compared to the control group (14.2 ± 0.4), the losartan group (11.6 ± 1.2), and the copper combined with losartan group (11.2 ± 0.9). Additionally, the copper-treated group exhibited higher gene expression of the *TLR4* receptor (control: 1.00 ± 0.2; Cu: 1.86 ± 0.2; Los: 0.99 ± 0.2; Cu + Los: 0.48 ± 0.13) and *arginase-1* (control: 1.00 ± 0.22; Cu: 2.27 ± 0.4; Los: 0.75 ± 0.2; Cu + Los: 0.4 ± 0.17) compared to the other investigated groups. For the *F4/80* gene, the copper-treated group showed significantly different values compared to the control and losartan groups, but no difference was observed when compared to the copper combined with losartan group (control: 0.97 ± 0.04; Cu: 1.25 ± 0.06; Los: 0.81 ± 0.08; Cu + Los: 0.99 ± 0.18) (Figure 5).

## 4. Discussion

In the present study, our main findings indicate that administering twice the recommended copper dose to rats led to a shift in mesenteric PVAT toward a pro-inflammatory profile. This was characterized by the upregulation of inflammatory markers related to vascular regulation. These events appear to occur, at least in part, alongside adipose tissue renin–angiotensin system remodeling, with the involvement of the AT1R–TLR4–angiotensin II signaling pathway.

Despite the essential physiological and nutritional roles of copper, when its normal limit consumption is exceeded, toxic effects have been described in the neurological, cardiovascular, liver, and kidney cells [31,32,33]. Furthermore, we previously demonstrated that rats treated with high copper concentration exhibited toxic effects on cardiac and vascular tissues, impairing vascular reactivity, increasing blood pressure, and causing structural modifications of papillary muscles [10,11,12]. More recently, we provided some of the first evidence of copper-induced alterations in PVAT, marked by increased TNF-α production in thoracic depots [11]. Although the precise mechanisms of copper toxicity remain unclear, increased levels of ROS, oxidative stress, inflammation and disruptions in endocrine function have been identified as contributing factors [34,35,36,37].

Here, we expand on these findings, demonstrating that adipose tissue is a target of toxicity induced by chronic copper overload, which affects its secretory function (endocrine/paracrine/autocrine), revealing a new role for copper as a modulator of mesenteric PVAT. Copper-induced PVAT inflammation was evidenced, among others, by adipocyte hypertrophy, increased TNF-α and PAI-1 levels, and reduced secretion of the anti-inflammatory cytokine IL-10. Additionally, the gene expression of *MCP-1, TLR4*, and the total macrophage marker *F4/80* was upregulated, along with the increased expression of the M1 macrophage marker *CD86*.

Under conditions that promote chronic adipose tissue inflammation, adipocytes become hypertrophic, accompanied by significant infiltration of pro-inflammatory molecules [38]. Inflamed PVAT contributes to the progression of cardiovascular disease by increasing the expression of vasoregulatory pro-inflammatory adipokines, including primarily TNF-α, leptin, and MCP-1, and dysregulating the renin–angiotensin system, as well as decreasing the secretion of anti-inflammatory adipokines, such as adiponectin and IL-10 [15,24,25,26,38,39,40,41]. Dysfunctional adipocytes and M1 macrophages infiltrated into PVAT release TNF-α, which exerts atherogenic effects and promotes vascular dysfunction, insulin resistance, and dyslipidemia [42].

Along the same lines, PAI-1 is produced by adipose tissue and endothelial cells, playing a significant role in inflammation processes that negatively impact vascular biology, including the regulation of fibrinolysis and the downregulation of glucose and lipid metabolism [40,43,44,45].

Supporting our findings that copper treatment increased TLR4, previous studies have shown that copper can directly promote cardiotoxic effects and increase pro-inflammatory cytokines by acting as a ligand for TLR4, inducing the NF-κB and MAPK pathways in rat cardiac cells [34,46]. TLRs interact with both endogenous and exogenous molecules, regulating inflammatory response in adipose tissue, including PVAT [44]. TLR4-NF-κB signaling activates inflammatory genes like TNF-α, creating a cycle that intensifies inflammation in adipose tissue, primarily mediated by M1 macrophages [47,48,49].

Interestingly, the mesenteric PVAT of copper-treated animals showed a concomitant increase in antagonistic enzymes such as iNOS and arginase-1. iNOS is an enzyme that catalyzes the production of NO from L-arginine, a crucial molecule in cell signaling and vasodilation. The expression of iNOS is increased in PVAT in response to inflammatory stimuli, such as TNF-α and endotoxins. It plays a role in metabolic dysfunction, affecting glucose and lipid metabolism, mitochondrial activity, and reducing anti-inflammatory cytokine levels [44,50,51,52].

On the other hand, adipocytes also secrete arginase, an enzyme that reduces NO production by degrading L-arginine [53]. Increased arginase-1 activity, which can be triggered by ROS, TNF-α, and angiotensin II through AT1R [54,55], has been associated with vascular dysfunction in conditions such as hypertension, and acute myocardial infarction. This occurs through a NO-dependent mechanism by reducing arginine bioavailability [55,56,57,58,59]. Yao and colleagues (2022) demonstrated that, in chronic inflammatory conditions, such as obesity, there is an increase in arginase-1 expression in visceral adipose tissue, accompanied by elevated levels of inflammatory cytokines like TNF-α, MCP-1, IL-10, VCAM-1, and ICAM-1, along with reduced NO bioavailability and consequent vascular dysfunction [60].

As previously discussed, macrophages, derived from circulating monocytes, represent the most abundant immune cell population in adipose tissue. Alterations in their phenotype, particularly polarization toward a pro-inflammatory M1 profile, have been implicated in PVAT dysfunction and vascular inflammation, characterized by enhanced secretion of TNF-α and IL-6 and upregulation of iNOS [47,48,49]. In addition to macrophages, T lymphocytes also contribute to the regulation of inflammation and metabolic homeostasis in adipose tissue. The crosstalk between these immune cells within the unique adipose microenvironment is established as a modulator of inflammatory cascades [61]. However, T cell responses were not evaluated in the present study and should be considered in future investigations.

Another key finding of this study is that copper overload appears to trigger inflammatory signaling mechanisms involving the RAS. This is evidenced by the increase in serum angiotensin II, along with the upregulation of *ACE1* and *AT1R* gene expression in the mesenteric PVAT of copper-treated rats. Components of the RAS, such as angiotensinogen, renin, ACE1, and ACE2, as well as AT1R and Mas receptors, are synthesized by both white and brown adipocytes and respond to circulating angiotensin II through AT1R and AT2R [7,23,61,62,63]. It is suggested that RAS activity in PVAT plays a crucial role in vascular regulation [62,63]. However, under pathological conditions, angiotensin II binding to AT1R can activate pro-inflammatory signaling pathways, leading to PVAT dysfunction and consequent vascular damage [64].

It is well established that the inflammatory action of Ang II on adipocytes is mediated by AT1R stimulation. Specifically, Ang II binds to AT1R and activates NADPH oxidase, increasing ROS production, which in turn activates the NF-κB pathway, leading to the transcription of pro-inflammatory genes such as PAI-1, resistin, and MCP-1, thereby creating a pro-inflammatory environment that contributes to vascular and metabolic dysfunctions [48,62,65,66,67,68].

Furthermore, it has been described that the action of Ang II also can be mediated by TLR4 stimulation, which culminates in the activation of the JNK/NF-κB signaling cascades, leading to an increase in pro-inflammatory cytokines and the generation of ROS in a MyD88-dependent manner [65,66,67,68]. The link between TLR4 and the RAS was also demonstrated in a study with 3T3-L1 adipocytes, showing that TLR4/NF-κB inflammatory signaling is associated with the hyperactivation of local RAS [69].

It is important to note that RAS in adipose tissue is not only a regulator of endocrine function but also a key regulator of adipocyte metabolism [62]. Consistent with our findings of reduced unstimulated lipolysis and adipocyte hypertrophy induced by copper overload, Ang II promotes lipid storage in adipocytes. Its binding to AT1R inhibits adipogenesis and lipolysis. This antilipolytic effect of Ang II has been partially linked to reduced local blood flow in white adipose tissue. Additionally, Ang II interacts with AT2R, which enhances the activity of key enzymes involved in lipid synthesis, thus promoting lipogenesis [62,70].

In an attempt to enhance the significance and relevance of these results regarding the secretory function of mesenteric PVAT and its pro-inflammatory profile induced by copper overload, we next tested the hypothesis that the increase in vascular regulation-related inflammatory markers involves mechanisms associated with the upregulation of AT1R in the PVAT. Accordingly, we treated Wistar rats with copper overload for 30 days, combined with losartan, a pharmacological AT1R antagonist.

In fact, unlike copper treatment alone, the combination of copper treatment with losartan did not result in an increase in TNF-α, TLR4, and ARG1. Additionally, no significant increase in the diameter of mesenteric PVAT adipocytes was detected, indicating that the blockade of AT1R signaling mitigated the pro-inflammatory effects induced by copper overload in PVAT.

Corroborating our findings, studies have consistently shown that blocking AT1R signaling can reduce the production of pro-inflammatory cytokines and modulate inflammation in various models. For instance, in a mouse model of cardiac dysfunction induced by sepsis, pre-treatment with losartan reversed the increase in IL-1β, IL-6, TNF-α, and MCP-1 [68]. Similarly, De Batista et al. demonstrated that losartan administration attenuated TLR4 expression in SHR animals [71]. Additionally, Shatanawi et al. found that AT1R blockade prevented the increase in arginase activity in endothelial cells [55].

**Figure 6:**
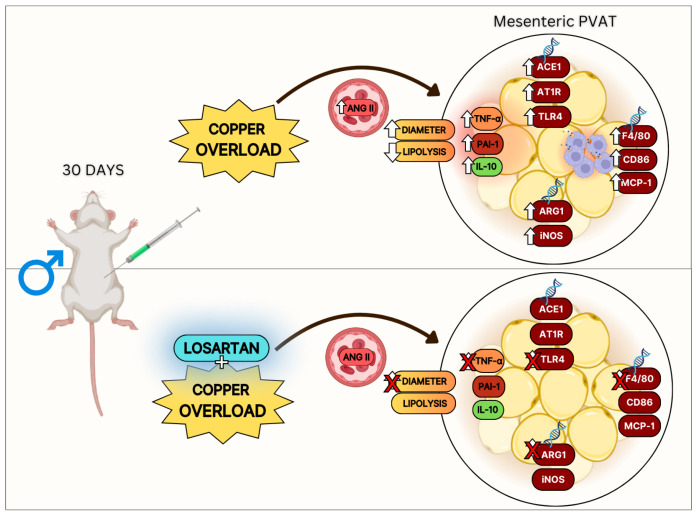
Summary of the effects of chronic copper overload and copper overload combined with losartan treatment on mesenteric PVAT in Wistar rats. Ang II, angiotensin II; AT1R, angiotensin II type 1 receptor; PVAT, perivascular adipose tissue; TLR4, Toll-like receptor 4; NF-κB, nuclear factor kappa B; MCP-1, monocyte chemoattractant protein-1; ACE1, angiotensin-converting enzyme 1; ARG1, arginase-1; iNOS, inducible nitric oxide synthase; IL-10, interleukin-10; PAI-1, plasminogen activator inhibitor type 1; CD86, cluster of differentiation 86; TNF-α, tumor necrosis factor alpha.

Therefore, our findings provide novel evidence that chronic copper overload induces mesenteric PVAT dysfunction, characterized by increased expression of inflammatory adipokines and vasoregulatory markers, occurring alongside alterations in the local RAS, particularly the AT1R–TLR4–angiotensin II signaling pathway. Notably, AT1R blockade with losartan appears to mitigate these effects, reducing inflammation and preventing adipocyte hypertrophy. These results reinforce the role of RAS as an important autocrine regulator of PVAT function and suggest that excessive copper intake may contribute to metabolic and vascular disturbances through inflammatory mechanisms (Figure 6).

Considering the growing interest in the impact of trace metals on adipose tissue physiology, our study provides new insights into copper’s role in PVAT modulation and its interplay with PVAT inflammation, opening perspectives for future research on potential therapeutic strategies targeting the PVAT RAS in metabolic and cardiovascular diseases.

Furthermore, our findings underscore the need to consider the impact of non-recommended copper doses on PVAT function, particularly regarding its potential contribution to vascular and metabolic disorders. This highlights an important public health concern, namely excessive copper intake, especially through unsupervised supplementation.

Despite the relevance and novelty of the data presented in this study, our experimental protocol has some limitations. Although a range of molecular alterations were observed, the causal relationships between copper overload and signaling pathways such as TLR4 or AT1R, as well as the underlying mechanisms involved, still require further exploration. It was not possible to determine whether copper acts as a direct ligand for TLR4 or any other inflammatory signaling pathway in PVAT. In addition, the data presented here were obtained from whole PVAT analysis, considering that this tissue is composed of a conglomerate of different cell types, including adipocytes, preadipocytes, immune cells, and mesenchymal stem cells, embedded in a matrix rich in microvessels. Therefore, we could not specifically identify which of these cells were primarily affected by copper overload toxicity. Future studies are necessary to gain a deeper understanding of the roles played by distinct cell types in this context.

## 5. Conclusions

In conclusion, our study demonstrates that chronic exposure to double the recommended copper dose disrupts the secretory function of mesenteric PVAT, promoting inflammation and altering the local RAS. These effects appear to occur, at least partially, alongside AT1R-TLR4 interactions, contributing to the upregulation of inflammatory and vasoregulatory markers. Importantly, AT1R blockade prevents these detrimental changes, reinforcing the significance of RAS modulation in PVAT homeostasis.

## Figures and Tables

**Table 1:** Forward (sense) and reverse (antisense) sequences of the primers used in qPCR.

Gene	5′ Primer (5′–3′)	3′ Primer (5′–3′)
*ACE1*	CCACGTCCCGGAAATACGAA	CCTGCATCAGAGTAGCCGTT
*ACE2*	GCCCAAAAGATGAACGAGGC	GACGCTTGATGGTCGCATTC
*Angiotensinogen*	CATCTTCACCCCCTCGAGTA	TGCTTCTGTGTGTCCTTTAGC
*Arg1*	GGTGGAGACCACAGTATGGC	GCAGATTCCCAGAGCTGGTT
*AT1R*	GGATTCGTGGCTTGAGTCCT	CGAAATCCACTTGACCTGGTG
*AT2R*	TGCTCTGACCTGGATGGGTA	AGCTGTTGGTGAATCCCAGG
*CD206*	GGAGGGTGCGGTACACTAAC	GTAGCCGGGATTTCGTCTGA
*CD86*	AAGACATGTGTAACCTGCACCA	AAGCTTGCCTCTTCACAGGA
*F4/80*	GGACAAAGACTTAACGGTGTGA	TGCTGGGCAGAAAACCTTGT
*B-actin*	ACACCCGCCACCAGTTCG	CCCACGATGGAGGGGAAGAC
*Leptin*	ATTTCACACACGCAGTCGGT	CCAGGGTCTGGTCCATCTTG
*MAS1*	GGAAGACCAGCCCACAGTTAC	ATCACAGGAAGAGAGCCTCG
*MCP-1*	TGTCTCAGCCAGATGCAGTT	CAGCCGACTCATTGGGATCA
*NOS 2/iNOS*	GGTGAGGGGACTGGACTTTT	TTCTCCGTGGGGCTTGTAGT
*TLR4*	TCTGAGCTTCAACCCCCTGA	TTGTCTCAATTTCACACCTGGA
*TLR9*	CCATTTTCCATCATGGTTCTCTG	GCCATGAGGCTTCAGTTCAC

Font: https://www.ncbi.nlm.nih.gov/tools/primer-blast, accessed on 3 November 2023.

**Table 2:** Body weight gain, food intake, feed efficiency, adiposity, mesenteric PVAT adipocyte diameter, and biochemical analysis of Wistar rats treated for 30 days with daily intraperitoneal injections of saline (control) or copper (25.72 µg/kg/day).

	Control	Copper
Body weight gain (g)	89 ± 7.9	97 ± 9.8
Feed efficiency (g/g)	0.14 ± 0.007	0.15 ± 0.009
Food intake (g/day)	29.3 ± 0.5	27.1 ± 0.4 *
Blood glucose (mg/dL)	104 ± 6.1	105 ± 4.7
Total cholesterol (mg/dL)	75 ± 3	81 ± 5.7
Triglyceride (mg/dL)	122 ± 12.7	126 ± 13.2
Inguinal WAT (mg/g BW)	12.1 ± 0.4	11.8 ± 0.8
Visceral WAT (mg/g BW)	8.6 ± 0.6	8.8 ± 1.1
Mesenteric Adipocyte diameter (µm)	48.3 ± 2	58.9 ± 3.2 *

Values are means ± SEM; n = 8 per group. * *p* < 0.05 vs. control. Student’s *t*-test. BW, body weight; WAT, white adipose tissue.

**Table 3:** Body weight gain, food intake, feed efficiency, adiposity, mesenteric PVAT adipocyte diameter, and biochemical analysis of Wistar rats treated for 30 days with daily intraperitoneal injections of saline (control), copper (25.72 µg/kg/day), losartan (10 mg/kg), and copper combined with losartan (25.72 µg/kg/day + 10 mg/mL).

	Control	Copper	Losartan	Copper + Losartan
Body weight gain (g)	110 ± 9.6	121 ± 8.5	108 ± 3.3	104 ± 4.3
Feed efficiency (g/g)	0.11 ± 0.006	0.10 ± 0.007	0.10 ± 0.007	0.11 ± 0.01
Food intake (g/day)	29.8 ± 1.2	28 ± 1.1 *	28.7 ± 0.8	28.8 ± 0.9
Blood glucose (mg/dL)	103 ± 1.7	108 ± 6.6	102 ± 7.4	100 ± 5
Total cholesterol (mg/dL)	99 ± 3.8	98 ± 4.9	87 ± 5.4	101 ± 5.7
Triglyceride (mg/dL)	90 ± 9.1	112 ± 21	88 ± 10	99 ± 24
Inguinal WAT (mg/g BW)	11.3 ± 0.5	11.9 ± 0.7	10.6 ± 0.8	10.5 ± 0.9
Visceral WAT (mg/g BW)	8.1 ± 1.1	8.2 ± 0.9	8.5 ± 0.7	8.4 ± 0.4
Mesenteric adipocyte diameter (µm)	41.3 ± 0.5	49.9 ± 0.7 *	41.0 ± 0.8	44.2 ± 1.3

Data are expressed as mean ± SEM. * *p* < 0.05 vs. control. n = 6 per group. One-way ANOVA followed by Tukey’s post hoc test. WAT, white adipose tissue. BW, body weight.

## Data Availability

The original contributions presented in this study are included in the article. Further inquiries can be directed to the corresponding author.

## Abbreviations

ACE1/2	Angiotensin II type 1 receptor
ARG1	Arginase-1
AT1R	Angiotensin-converting enzyme 1/2
CU	Copper
Los	Losartan
NO	Nitric oxide
PVAT	Perivascular adipose tissue
RAS	Reactive oxygen species
ROS	Renin–angiotensin system
